# Sarcopenia-related gut microbiota in the elderly: Insights from the longevity region of Kyotango and its nutritional associations

**DOI:** 10.1080/29933935.2025.2591561

**Published:** 2025-12-09

**Authors:** Takeshi Yasuda, Tomohisa Takagi, Yuji Naito, Ryo Inoue, Katsura Mizushima, Kouhei Asaeda, Hikaru Hashimoto, Hiroaki Kitae, Kazuhiko Uchiyama, Norihiro Ouchi, Atsuo Adachi, Tadaaki Kamitani, Satoaki Matoba, Yoshito Itoh

**Affiliations:** a Department of Molecular Gastroenterology and Hepatology, Graduate School of Medical Science, Kyoto Prefectural University of Medicine, Kyoto, Japan; b Department of Molecular Gastroenterology and Hepatology/Department for Medical Innovation and Translational Medical Science, Graduate School of Medical Science, Kyoto Prefectural University of Medicine, Kyoto, Japan; c Department of Human Immunology and Nutrition Science, Kyoto Prefectural University of Medicine, Kyoto, Japan; d Department of Applied Biological Sciences, Faculty of Agriculture, Setsunan University, Osaka, Japan; e Department of Internal Medicine, Kyotango Municipal Yasaka Hospital, Kyoto, Japan; f Department of Cardiovascular Medicine/Department of Longevity and Regional Epidemiology, Graduate School of Medical Science, Kyoto Prefectural University of Medicine, Kyoto, Japan

**Keywords:** Sarcopenia, gut microbiota, Japanese dietary culture, Japanese diet index, butyrate-producing bacteria

## Abstract

Sarcopenia is influenced by the gut microbiota and dietary habits; however, the underlying mechanisms remain elusive. This study investigated the gut microbiota composition of elderly individuals in a healthy longevity region and examined its association with sarcopenia and dietary habits. Fecal metagenomic analysis was used to identify gut microbiota taxonomy. Sarcopenia was diagnosed on the basis of grip strength, gait speed, and muscle volume. Japanese dietary habits were assessed using a brief-type self-administered diet history questionnaire. A total of 318 elderly individuals from Kyotango were recruited, 5.7% of whom were diagnosed with sarcopenia. Individuals with sarcopenia exhibited a lower abundance of a genus belonging to the family *Lachnospiraceae*, and a higher abundance of *Megasphera*. Several butyrate-producing bacteria, including *Lachnospira* and *Coprococcus* showed a positive correlation with sarcopenia related factors, whereas *Dorea* and *Streptococcus* were negatively correlated. Hierarchical cluster analysis revealed that these beneficial genera were also positively associated with the frequent intake of traditional Japanese dietary components. These findings suggest that the observed microbial and dietary associations may provide a mechanistic basis for potential protective effects against sarcopenia. Our findings suggest that butyrate-producing bacteria associated with Japanese dietary patterns play a protective role against sarcopenia.

## 1. Introduction

In recent years, the global population has experienced an accelerated aging trend. Specifically, the Japanese mean lifetime consistently ranks among the world's highest and continues to increase annually. Nevertheless, healthy life expectancy has not increased.^1^ The causes of this gap include sarcopenia, unhealthy dietary habits, hypertension, smoking, obesity, and diabetes.^2‐5^ Sarcopenia is typically diagnosed using muscle strength indicators, such as grip strength and gait speed, in addition to total body skeletal muscle volume.^6^

A Japanese cohort study on the general elderly population published in 2021 showed that the prevalence of sarcopenia was 11.5% in men and 16.7% in women.^7^ The prevalence of sarcopenia increases with age; approximately 22% of men and women in the 75–79 age group and 32% of men and 48% of women over 80 years of age have sarcopenia. Notably, sarcopenia has been shown to increase the risk of both death and long-term care by approximately two-fold, thereby undermining healthy longevity.^7^ However, the pathogenesis of sarcopenia is complex and yet to be elucidated.

The association between sarcopenia and the gut microbiota has been reported in recent years.^8‐11^ A systematic review of the relationship between gut microbiota, muscle volume, and physical performance suggested that a decrease in butyrate-producing bacteria, such as Clostridium XIVa and Roseburia, promotes anabolic resistance and chronic inflammation, which may be associated with muscle atrophy.^8^ Another study also demonstrated that several butyrate producers, including *Lachnospira*, *Prevotella*, *Ruminococcus*, and *Clostridium*, were reduced in individuals with sarcopenia.^9^^,^^10^ Furthermore, Han et al. reported that elderly people with decreased muscle mass exhibited a lower proportion of Bacillota compared to Bacteroidota.^11^

Daily dietary habits also influence sarcopenia onset. The Mediterranean diet, which is known to promote health and longevity, is associated with a lower incidence of sarcopenia among individuals who consume it frequently.^12^ Similar to the Mediterranean diet in Europe, the traditional Japanese diet is known for its emphasis on healthy food.^13^ A previous study investigated the relationship between the frequency of Japanese diet consumption and grip strength ^14^ and found that individuals with a decline in grip strength tended to consume the Japanese diet less frequently.

The Kyotango area (Kyotango City, Miyazu City, Yosano Town, and Ine Town, located in the northernmost part of Kyoto Prefecture) is a region with traditional Japanese dietary culture. This region has gained attention as the healthiest and longest-living area in Japan.^15^ In the Kyotango area, the ratio of centenarians is approximately three times the national average (per 100,000 people) (as of March 2020, the Japanese Basic Resident Ledger). The longevity of residents in this area has been suggested to be attributed to the prevalent Japanese dietary culture. Interestingly, the residents of this region have a higher prevalence of butyrate-producing bacteria in their intestines than residents of urban areas.^15^

While previous studies have reported an association between reduced butyrate-producing bacteria and sarcopenia,^8‐10^ few have focused on cohorts from longevity regions with distinctive traditional dietary habits. In particular, there is a lack of research investigating how dietary patterns unique to the Japanese culture may influence the abundance of specific butyrate-producing genera and their relationship with muscle function.

Building on the background of the traditional Japanese dietary culture and longevity of the Kyotango region, this study aimed to investigate the interplay between sarcopenia and its indicators (grip strength, gait speed, and skeletal muscle volume) and the gut microbiota in an elderly cohort of Kyotango residents. Furthermore, a comprehensive dietary intake survey focusing on this unique Kyotango dietary culture was conducted.

## 2. Methods

### 2.1. Participants and sarcopenia diagnosis

Faecal metagenome analysis was performed on elderly individuals aged 65 years or older who underwent community health checkups in the Kyotango area. The results were integrated with various data obtained from medical examinations conducted during health checkups to clarify the factors related to sarcopenia, faecal gut microbiota, and their relationship with diet.

**Figure 1.**
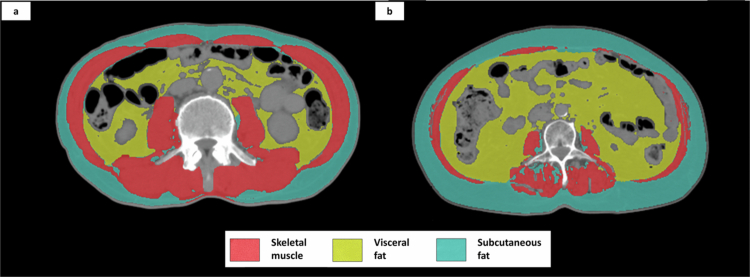
Examples of the distribution of skeletal muscle, visceral fat, and subcutaneous fat in the L3 section of abdominal CT in a) non-sarcopenia and b) sarcopenia cases.

Sarcopenia was diagnosed based on the Asian Working Group for Sarcopenia (AWGS) diagnostic criteria, which include grip strength, gait speed, and skeletal muscle volume.^6^ The cut-off values for grip strength were set at 28 kg for men and 18 kg for women, and the cut-off values for gait speed were set at 1.0 m/s for both men and women.^6^ Skeletal muscle mass is commonly measured using dual-energy X-ray absorptiometry (DEXA) and bioelectrical impedance analysis (BIA) methods. The skeletal muscle volume in the L3 cross-section of the abdominal CT correlates well with an individual's total body skeletal muscle volume.^16^ Therefore, we calculated the Skeletal Muscle mass (SMI) index from abdominal CT-L3 cross-sections using the Slice Omatic® (Tomo Vision) software (Figure 1). The cut-off values of SMI were 42 cm^2^/m^2^ for men and 38 cm^2^/m^2^ for women, based on the diagnostic criteria of the Japan Society of Hepatology.^16^

### 2.2. Assessment of dietary intake

**Figure 2.**
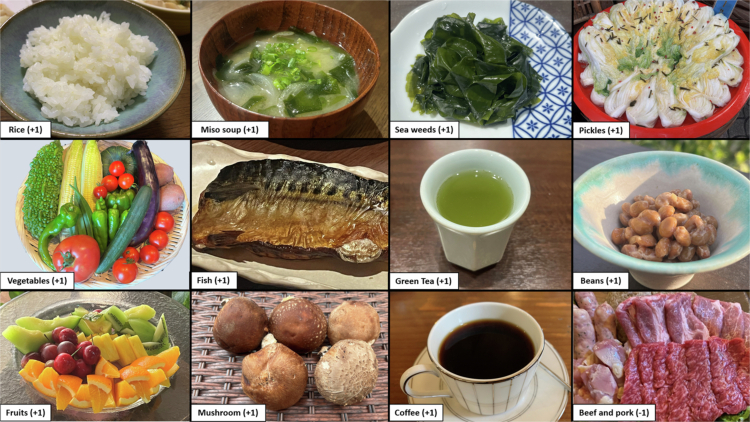
The Japanese diet components included in r JDI−12.

A brief-type self-administered diet history questionnaire (BDHQ) was used to assess dietary and nutrient intakes.^17^ The raw data obtained from the BDHQ were adjusted for energy using the density method. The following nutritional components that can be calculated from the BDHQ were selected: three major nutrients (protein (animal- and plant-derived), lipids (animal- and plant-derived), and carbohydrates), vitamin D, and dietary fibre (soluble and insoluble), which are known to be associated with sarcopenia or maintenance of health.^18‐22^ The Japanese Diet Index (JDI) was used to calculate traditional Japanese cuisine intake.^13^ Based on the revised JDI−12 (r JDI−12) reported by Saji et al., we added points for rice, miso soup, seaweeds, pickles, green and yellow vegetables, fish, green tea, beans, fruits, mushrooms, and coffee, and subtracted points for beef and pork (Figure 2).^23^ The added and subtracted points were based on the median value of the intake of each dietary component calculated from the BDHQ, after adjusting for energy using the density method.

### 2.3. Analysis of gut microbiota

Examination of the faecal gut microbiota was performed using the same methodology as in our previous study.^24^ Genomic DNA was extracted using the NucleoSpin Microbial DNA Kit (Macherey-Nagel, Düren, Germany). The 16S rRNA gene contains nine variable regions (V1-V9), of which the V3-V4 regions were amplified by polymerase chain reaction. Nucleotide sequencing was performed using the MiSeq platform (Illumina, San Diego, CA, USA). The sequence data were examined using the Quantitative Insights into Microbial Ecology 2 (QIIME 2). To process the sequence reads, the DADA2 model was employed for denoising, which resulted in the identification of amplicon sequence variants (ASV) and representative sequences.^25^^,^^26^ Forward and reverse reads were trimmed at the first 17 and 19 bases, respectively, and truncated at positions 260 and 240 bp according to the Phred quality profiles. Low-quality reads with expected errors >2 or Phred quality <2 were automatically filtered out, and chimeric sequences were removed using the consensus method. ASVs observed fewer than two times across all samples were discarded (singleton removal). Phylogenetic placement of ASVs was performed using SEPP against the Greengenes reference tree, and ASVs not placed in the reference tree were removed. The final denoised feature table contained a total of 25,567,099 high-quality sequences (median 65765.5 reads per sample; range 21,105–404,314).

### 2.4. Statistical analysis

Statistical analyses were performed using JMP Pro version 15.2 (SAS International Inc., Cary, NC, USA), GraphPad Prism 9.00 for Windows (GraphPad Software, La Jolla, California, USA), and SPSS software (version 25.0; IBM Japan, Ltd., Tokyo, Japan). Differences were considered statistically significant at *P*-value < 0.05. Data are expressed as mean ± standard deviation (SD) for continuous variables and as frequencies (percentages) for categorical variables. Comparisons between the sarcopenia and non-sarcopenia groups were made using the Mann–Whitney U test and Jonckheere-Terpstra test.

Spearman’s rank correlation coefficients were calculated to examine the relationships between genus-level bacterial abundances and three muscle-related indicators: grip strength, gait speed, and skeletal muscle index (SMI).

*P*-values were adjusted for multiple comparisons using the Benjamini–Hochberg false discovery rate (FDR) correction method.

## 3. Results

### 3.1. Diagnosis of sarcopenia

In the Kyotango region, 329 elderly individuals aged ≥ 65 years underwent medical examinations between August 2017 and March 2019 (Figure 2). From this cohort, those who could not provide faecal samples or did not undergo CT were excluded; the remaining 318 participants were included. The study finally included 127 men (39.9%) and 191 women (60.1%), with an average age of 73.3 years, an average body mass index (BMI) value of 23.3 kg/m², an average maximal gait speed of 2.02 m/s, an average handgrip strength of 28.9 kg, and an average SMI of 41.8 cm²/m² (Table 1).

**Figure 3.**
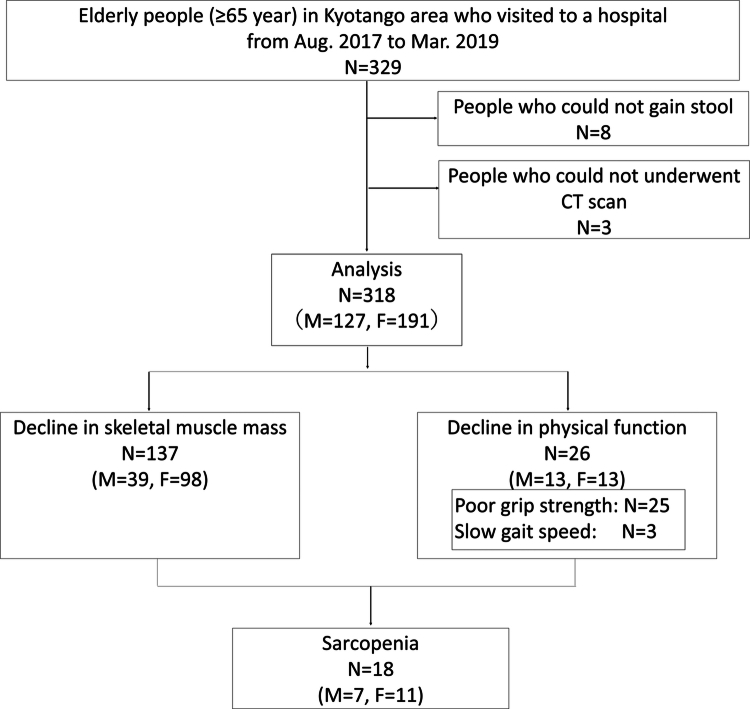
Flowchart depicting participant recruitment in this study.

Of the 318 participants, 137 exhibited a decline in muscular volume, 25 exhibited reduced grip strength, and three exhibited diminished gait speed (Figure 3). Two participants had decreased both grip strength and gait speed. Among these patients, sarcopenia was diagnosed in 18 (5.7%) patients (men 7, women 11) (sarcopenia group), whereas 300 (94.3%) patients did not have sarcopenia (non-sarcopenia group). This relatively low prevalence aligns with the overall health status of the region. However, the small sample size of the sarcopenia group may have affected the generalisability of our findings.

There were no significant differences in age, BMI, or gait speed between men and women. However, grip strength and SMI were significantly higher in men than in women (Table 1). The age was significantly higher in the sarcopenia group than in the non-sarcopenia group. The gait speed, grip strength, and SMI were significantly lower in the sarcopenia group than in the non-sarcopenia group. There were no significant differences in age or gait speed between men in the sarcopenia and non-sarcopenia groups. In contrast, age and gait speed were significantly higher and lower, respectively, in women with sarcopenia than in those without sarcopenia. Grip strength and SMI were significantly lower in both men and women in the sarcopenia group than in those in the non-sarcopenia group.

**Table 1.** Background of the participants.

a) Participant characteristics							
	Overall	Man	Woman	*P*-value	Non-sarcopenia	Sarcopenia	*P*-value
Number (%)	318	127 (39.9)	191 (60.1)	-	300 (94.3)	18 (5.7)	-
Age, mean (year, min, max)	73.3 (65, 101)	74.1 (65, 101)	72.7 (65, 89)	0.162	72.9 (65, 101)	78.7 (68, 88)	<0.001
BMI, mean (kg/m^2^, min, max)	23.3 (16.0, 38.5)	23.5 (16.5, 30.5)	23.2 (16.0, 38.5)	0.134	23.3 (16.0, 38.5)	23.6 (19.7, 31.1)	0.859
Gait speed (m/s, mean ± SD)	2.02 ± 0.40	2.07 ± 0.44	1.98 ± 0.36	0.053	2.04 ± 0.37	1.64 ± 0.60	0.004
Grip strength (kg, mean ± SD)	28.9 ± 8.10	36.4 ± 6.74	24.0 ± 4.26	<0.001	29.5 ± 7.90	19.4 ± 4.84	<0.001
SMI (cm^2^/m^2^, mean ± SD)	41.8 ± 7.77	47.1 ± 8.20	38.2 ± 5.60	<0.001	42.2 ± 7.82	35.7 ± 2.77	<0.001

b) Sex differences between sarcopenia and non-sarcopenia groups							
		Man			Woman		
	Overall	Non-sarcopenia	Sarcopenia	*P*-value	Non-sarcopenia	Sarcopenia	*P*-value
Number (%)	318	120 (94.5)	7 (5.5)	-	180 (94.2)	11 (5.8)	-
Age, mean (year, min, max)	73.3 (65, 101)	74.0 (65, 101)	74.9 (68, 85)	0.582	72.2 (65, 89)	81.2 (70, 88)	<0.001
BMI, mean (kg/m^2^, min, max)	23.3 (16.0, 38.5)	23.6 (16.5, 30.5)	22.6 (19.7, 25.4)	0.279	23.1 (16.0, 38.5)	24.2 (20.4, 31.1)	0.300
Gait speed (m/s, mean ± SD)	2.02 ± 0.40	2.07 ± 0.44	2.02 ± 0.43	0.987	2.02 ± 0.32	1.39 ± 0.57	<0.001
Grip strength (kg, mean ± SD)	28.9 ± 8.10	37.0 ± 6.28	24.7 ± 2.36	<0.001	24.5 ± 3.86	16.0 ± 2.00	<0.001
SMI (cm^2^/m^2^, mean ± SD)	41.8 ± 7.77	47.9 ± 7.07	37.9 ± 2.37	<0.001	38.4 ± 5.69	34.3 ± 2.09	0.006
							Mann–Whitney U test

BMI: body mass index; SMI: skeletal muscle mass index; SD: standard deviation.

### 3.2. Sarcopenia-related gut microbiota

**Figure 4.**
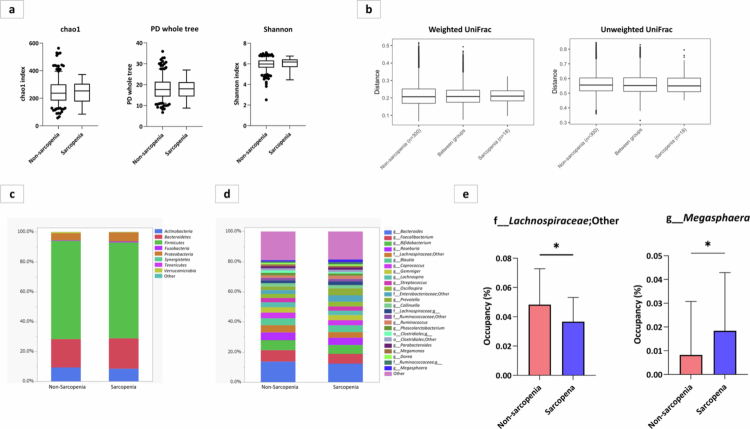
Presence of sarcopenia, diversity of gut microbiota, taxonomy of the gut microbiota, and significant differences in gut microbiota in participants with and without sarcopenia. a) Alpha diversity of the gut microbiota. The Chao1 index (*P*-value = 0.917), PD whole tree (*p* = 0.833), and Shannon indices (*p* = 0.344), did not exhibit substantial disparities between the sarcopenia and non-sarcopenia groups. b) *β* diversity of the gut microbiota. No difference was observed between the sarcopenia and non-sarcopenia groups (weighted UniFrac, *p* = 0.797; unweighted UniFrac, *p* = 0.642). c) Taxonomy of gut microbiota at the phylum level. d) Taxonomy of gut microbiota at the genus level. e) The sarcopenia group showed a significantly lower abundance of the genus belonging to the family *Lachnospiraceae* and a significantly lower abundance of the genus *Megasphaera*.

The faecal gut microbiota of the sarcopenia and non-sarcopenia groups was compared. Initially, we analysed the heterogeneity of the gut microbiota within each group. Alpha diversity, evaluated using the Chao1 index, PD whole tree, and Shannon indices, did not exhibit substantial disparities between the sarcopenia and non-sarcopenia groups (Figure 4a). Similarly, considering both weighted and unweighted measures, no difference in beta diversity was observed between the sarcopenia and non-sarcopenia groups (weighted UniFrac, *p* = 0.797; unweighted UniFrac, *p* = 0.642) (Figure 4b).

We then analysed the taxonomy of the gut microbiota at the phylum (Figure 4c) and genus (Figure 4d) levels. Phylum *Firmicutes*, *Bacteroidetes*, *Actinobacteria*, and *Proteobacteria* were the four major phyla and showed no difference in their abundances in both the sarcopenia and non-sarcopenia groups. At the genus level, 35 extractable bacteria had an occupancy rate of >0.5%. The three most abundant genera in both groups were the genus *Bacteroides*, *Faecalibacterium,* and *Bifidobacterium.*

Furthermore, patients with sarcopenia exhibited a significantly lower abundance of an unclassified genus belonging to the family *Lachnospiraceae,* and a significantly higher abundance of *Megasphaera* (Figure 4e). Notably, *Lachnospiraceae* includes several butyrate-producing bacteria that have been linked to anti-inflammatory effects and energy metabolism.

### 3.3. Gut microbiota associated with sarcopenia indicators

We determined the association of gut microbiota with sarcopenia indicators (grip strength, gait speed, and SMI). The correlations (Spearman's rank correlation coefficient) between these indicators for sarcopenia diagnosis and the gut microbiota are presented in Table 2. Among the 35 genera with abundances >0.5%, we identified 10 with occupancy rates that suggested potential positive or negative correlations with grip strength, gait speed, or muscle mass. Those with a *P*-value ≥ 0.05 were excluded.

**Table 2.** The correlations between sarcopenia-related diagnostic indicators and the gut microbiota.

a) Correlation between grip strength and gut microbiota				b) Correlation between gait speed and gut microbiota				c) Correlation between muscle volume and gut microbiota			
Man				Man				Man			
Positive correlation	r	*P*-value	FDR-p	Positive correlation	r	*P*-value	FDR-p	Positive correlation	r	*P*-value	FDR-p
f__*Lachnospiraceae*;Other	0.271	0.002	0.080	g__*Coprococcus*	0.233	0.009	0.229	f__*Lachnospiraceae*;Other	0.219	0.013	0.517
g__*Lachnospira*	0.180	0.043	0.515	g__*Gemmiger*	0.215	0.017	0.229				
Negative correlation	r	*P*-value	FDR-p	Negative correlation	r	*P*-value	FDR-p	Negative correlation	r	*P*-value	FDR-p
g__*Streptococcus*	−0.179	0.044	0.515	g__*Ruminococcus*	−0.213	0.018	0.229	None			
Woman				Woman				Woman			
Positive correlation	r	*P*-value	FDR-p	Positive correlation	r	*P*-value	FDR-p	Positive correlation	r	*P*-value	FDR-p
None	―	―	―	g__*Phascolarctobacterium*	0.143	0.049	0.794	None	―	―	―
Negative correlation	r	*P*-value	FDR-p	Negative correlation	r	*P*-value	FDR-p	Negative correlation	r	*P*-value	FDR-p
g__*Megasphaera*	−0.211	0.003	0.133	g__*Streptococcus*	−0.191	0.008	0.319	*f__Lachnospiraceae;g__*	−0.160	0.027	0.935
g__*Lactobacillus*	−0.151	0.037	0.523								

FDR-P: False Discovery Rate-corrected *p.* r: Spearman's rank correlation coefficient.

In men, the genus *Lachnospira* and an unclassified genus belonging to the family *Lachnospiraceae* were positively correlated, while the genus *Streptococcus* was negatively correlated with grip strength. In women, the genera *Megasphaera* and *Lactobacillus* were negatively correlated with grip strength.

Regarding gait speed, the genera *Coprococcus* and *Gemmiger* were positively correlated in men, whereas the genus *Ruminococcus* was negatively correlated. In women, the genus *Phascolarctobacterium* was positively correlated with gait speed, whereas the genus Streptococcus was negatively correlated.

As for the SMI, in men, an unclassified genus belonging to the family *Lachnospiraceae* was positively correlated*.* After applying FDR correction separately for each subgroup, no genus reached the significance threshold (FDR-adjusted *P* < 0.05).

### 3.4. Sarcopenia and daily food consumption

We investigated the correlation between sarcopenia and the intake of each nutritional component (protein, fat, carbohydrate, vitamin D, and dietary fibre, as shown in Section 2.2) calculated from the BDHQ (energy-adjusted value). These nutritional components were not significantly different between participants with and without sarcopenia (Table 3a).

Upon investigating the Japanese diet consumption calculated using r JDI−12, we identified some features of the intake pattern in the Kyotango area. Compared to the Japanese average, the Kyotango area exhibited a higher consumption of seaweed, fish, beans, vegetables, and fruits, whereas meat intake was notably low (Table 3b).^27‐29^ There was no significant difference in the frequency of Japanese dietary intake between the sarcopenia and non-sarcopenia groups. The r JDI−12 was slightly lower in the sarcopenia group than in the non-sarcopenia group; however, the difference was not statistically significant (sarcopenia group: non-sarcopenia group = 5.7:6.0, *p* = 0.56).

**Table 3.** Gut microbiota correlations with nutrients and 12 Japanese dietary components.

a) Association between sarcopenia and sarcopenia related nutrition (energy-adjusted value [g/day])					
		(mean ± SD)			
Nutrition (%E)	Japanese average*	Overall	Non Sarcopenia	Sarcopenia	*P*-value
Protein [g]	69.0	81.5 ± 18.0	81.8 ± 18.2	76.7 ± 13.9	0.215
Animal protein	37.3	48.7 ± 18.3	49.0 ± 18.4	43.4 ± 14.0	0.169
Plant protein	31.7	32.8 ± 6.2	32.8 ± 6.2	33.2 ± 6.5	0.940
Fat [g]	55.3	56.3 ± 12.8	56.4 ± 12.9	54.4 ± 12.2	0.529
Animal fat	28.0	26.7 ± 9.0	26.9 ± 9.0	24.0 ± 7.4	0.233
Plant fat	27.3	29.5 ± 8.2	29.5 ± 8.2	30.5 ± 8.1	0.733
Carbohydrate [g]	235.3	261.6 ± 40.4	260.9 ± 40.7	272.4 ± 34.7	0.311
Vitamin D [μg]	8.2	22.2 ± 13.1	22.3 ± 13.1	20.0 ± 11.9	0.372
Total dietary fibre [g]	19.8	14.4 ± 4.3	14.4 ± 4.3	14.1 ± 3.9	0.795
Soluble dietary fibre	4.0	3.6 ± 1.2	3.6 ± 1.2	3.5 ± 1.1	0.911
Insoluble dietary fibre	13.0	10.2 ± 2.9	10.2 ± 2.9	10.0 ± 2.7	0.850
					Mann–Whitney U test

b) Association between sarcopenia and amount of Japanese diet intake (energy-adjusted value [g/day])					
		(mean ± SD)			
Japanese diet [g] (%E)	Japanese average**	Overall	Non Sarcopenia	Sarcopenia	*P*-value
Rice	139.3	290.7 ± 143.2	290.5 ± 142.9	295.3 ± 152.3	0.742
Miso soup	ー	179.7 ± 154.9	180.8 ± 156.7	162.3 ± 124.1	0.728
Seaweeds	2.1	17.7 ± 16.0	17.8 ± 16.3	14.9 ± 9.3	0.961
Pickles	ー	49.2 ± 35.6	48.7 ± 35.4	57.6 ± 38.3	0.153
Green and yellow vegetables	71.7	117.1 ± 72.1	117.1 ± 72.5	116.1 ± 66.8	0.991
Green Tea	ー	284.5 ± 295.1	286.6 ± 292.0	250.2 ± 352.3	0.269
Beans	24.6	86.1 ± 58.6	87.0 ± 58.7	71.8 ± 57.7	0.187
Fish	60.3	106.3 ± 58.9	106.9 ± 59.1	97.0 ± 56.1	0.359
Fruits	90.9	161.1 ± 110.9	161.5 ± 113.1	154.4 ± 65.7	0.795
Mushroom	9.4	13.7 ± 12.2	13.8 ± 12.1	11.8 ± 12.8	0.399
Coffee	230.6***	250.2 ± 186.7	250.2 ± 188.7	251.7 ± 153.5	0.717
Beef and pork	52.8	30.2 ± 21.6	30.1 ± 21.7	33.2 ± 19.6	0.392
r JDI−12	ー	6.0 ± 2.0	6.0 ± 0.1	5.7 ± 2.0	0.559
					Mann–Whitney U test

* The National Health and Nutrition Survey in Japan, 2019, Ministry of Health, Labour and Welfare.

** Food supply and demand chart, 2022, Ministry of Agriculture, Forestry and Fisheries.

*** Basic Survey on Coffee Demand Trends, 2020, National Coffee Roasters Association of Japan.

### 3.5. Nutritional and food factors associated with sarcopenia-related gut microbiota

**Figure 5.**
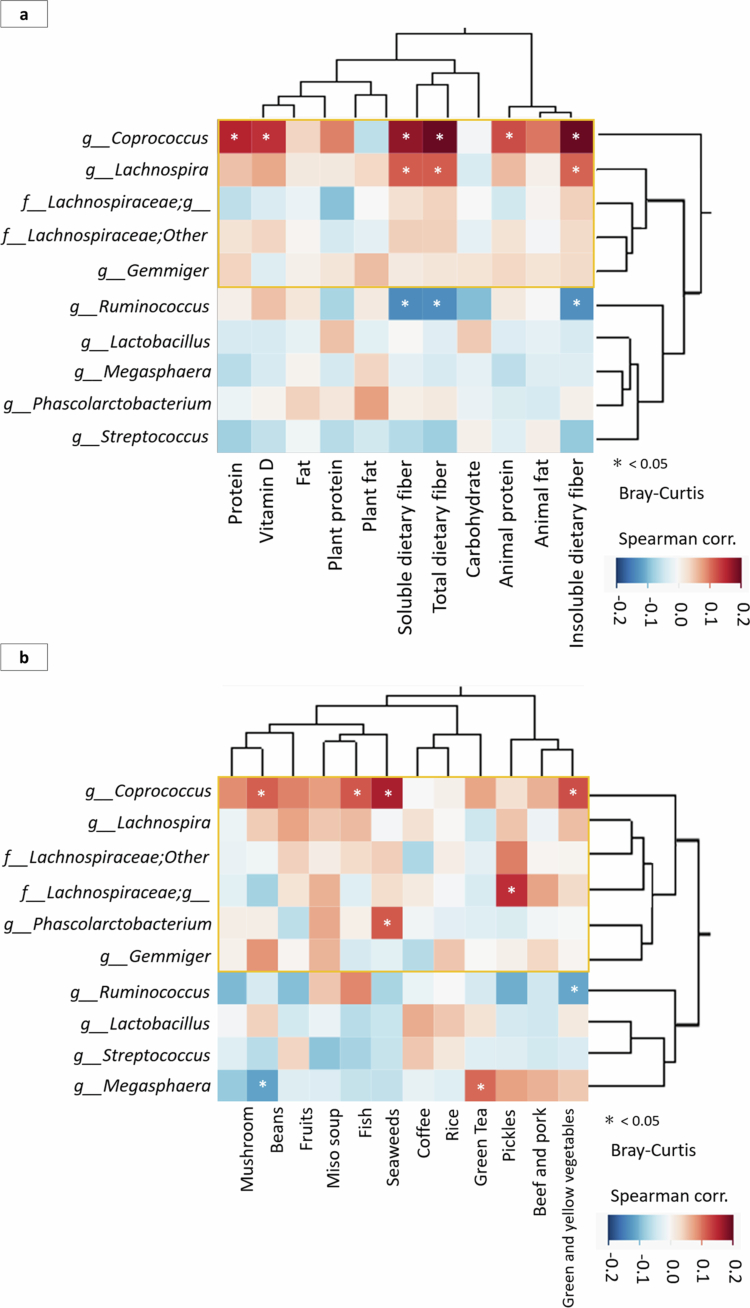
Hierarchical cluster analysis depicting a) the relationship between microbiota and nutrient intake, and b) the relationship between gut microbiota and 12 Japanese dietary components. Bray-Curtis dissimilarity was calculated from relative abundance data and used for hierarchical clustering. The heatmap displays the relative abundance of each genus across samples, clustered by microbiota similarity.

Hierarchical cluster analysis was performed to examine the relationship between the 10 genera associated with sarcopenia indicators (Table 2) and the 11 nutritional components calculated using the BDHQ (Figure 5a), as well as the consumption of the 12 Japanese dietary components (Figure 5b). Bray-Curtis dissimilarity was calculated from relative abundance data and used for hierarchical clustering. The heatmap displays the relative abundance of each genus across samples, clustered by microbiota similarity. We found that several genera, namely, *Coprococcus, Lachnospira,* an unclassified genus belonging to the family *Lachnospiraceae,* and *Gemmiger* were categorised as clusters that were positively correlated with nutritional components, especially protein, vitamin D, and dietary fibre (Figure 5a). The gut microbiota, which consisted of clusters that were positively correlated with each r JDI−12 component, was the same as the clusters that were positively correlated with nutrients associated with sarcopenia. Among these genera, *Coprococcus* —a representative butyrate-producing genus—, which was positively correlated with grip strength and gait speed, showed a significant positive correlation with the r JDI−12 score (Figure 5b).

### 3.6. Genus *Coprocossus* and the intake of Japanese dietary components

We assessed the association between the genus *Coprococcus* and the nutritional and Japanese dietary components using cluster analysis. The abundance of the genus *Coprococcus* was divided into quartiles, and the association with each nutrient or the Japanese diet (energy-adjusted value [g/day]) was analysed using the Jonckheere-Terpstra test (Table 4). The consumption of animal and plant proteins (*p =* 0.024 and *p =* 0.043, respectively), animal fat (*p =* 0.045), vitamin D (*p =* 0.014), and both soluble and insoluble dietary fibre (*p =* 0.002 and *p* < 0.001, respectively) was associated with a significant monotonic increase in the abundance of the genus *Coprococcus* (Table 4a). Furthermore, 12 Japanese dietary components, including seaweed (*p =* 0.006), beans (*p =* 0.01), fish (*p =* 0.021), green and yellow vegetables (*p =* 0.030), and mushrooms (*p =* 0.048), were associated with a significant monotonic increase in the abundance of *Coprococcus,* in addition to r JDI−12 (*p =* 0.001) (Table 4b).

**Table 4.** Associations of *Coprococcus* abundance with nutrients and 12 Japanese dietary components.

a) Genus *Coprococcus* and the intake of each nutrition (energy-adjusted value [g/day])					
		(median ± SD)			
	Trend test	g__*Coprococcus*			
	*P*-value	Q1	Q2	Q3	Q4
		0.0000−0.0179	0.0180−0.0314	0.0315−0.0531	0.0532−0.1900
Protein	**0.006**	76.9 ± 16.4	80.2 ± 16.6	84.4 ± 21.4	84.4 ± 16.2
Animal protein	**0.024**	44.3 ± 16.3	47.5 ± 16.3	52.5 ± 22.0	50.3 ± 17.0
Plant protein	**0.043**	32.6 ± 5.8	32.7 ± 7.7	32.0 ± 7.7	34.1 ± 5.1
Fat	0.412	56.2 ± 13.9	55.5 ± 12.7	56.0 ± 12.7	57.3 ± 12.1
Animal fat	**0.045**	25.5 ± 9.0	25.8 ± 8.5	27.7 ± 9.6	27.9 ± 8.7
Plant fat	0.340	30.7 ± 8.4	29.8 ± 8.6	28.3 ± 7.1	29.4 ± 8.7
Carbohydrate	0.940	264.1 ± 40.8	257.8 ± 41.5	262.5 ± 42.2	262.0 ± 37.4
Vitamin D	**0.014**	18.5 ± 8.9	22.0 ± 12.0	24.8 ± 16.9	23.5 ± 12.4
Total dietary fibre	**<0.001**	13.6 ± 4.6	14.0 ± 4.3	14.1 ± 3.9	15.8 ± 4.0
Soluble dietary fibre	**0.002**	3.4 ± 1.3	3.5 ± 1.3	3.5 ± 1.1	4.0 ± 1.1
Insoluble dietary fibre	**<0.001**	9.7 ± 3.2	10.0 ± 3.0	9.9 ± 2.6	11.2 ± 2.7
					Jonckheere-Terpstra test

b) Genus *Coprococcus* and the intake of each dietary item of the Japanese diet (energy-adjusted value [g/day])					
		(median ± SD)			
	Trend test	g__*Coprococcus*			
	*P*-value	Q1	Q2	Q3	Q4
		0.0000−0.0179	0.0180−0.0314	0.0315−0.0531	0.0532−0.1900
r JDI−12	**0.001**	5.6 ± 2.0	5.8 ± 2.1	6.0 ± 1.7	6.6 ± 1.9
Rice	0.437	288.7 ± 159.4	278.4 ± 135.9	304.7 ± 139.2	291.1 ± 138.6
Miso soup	0.205	178.7 ± 136.7	159.6 ± 149.5	151.3 ± 114.1	229.8 ± 198.1
Seaweeds	**0.006**	14.1 ± 11.7	15.6 ± 13.0	21.1 ± 21.6	19.9 ± 15.1
Pickles	0.455	48.4 ± 33.2	51.3 ± 41.6	47.2 ± 33.2	50.1 ± 34.2
Green and yellow vegetables	**0.030**	107.1 ± 70.4	119.5 ± 77.5	111.2 ± 66.1	130.8 ± 73.0
Fish	**0.021**	93.1 ± 47.1	103.1 ± 50.5	120.5 ± 78.2	108.6 ± 52.2
Green Tea	0.052	306.3 ± 357.2	215.3 ± 238.0	297.7 ± 292.6	319.4 ± 275.0
Beans	**0.010**	76.7 ± 59.8	90.8 ± 65.8	81.9 ± 50.7	95.0 ± 56.6
Fruits	0.059	146.8 ± 117.7	168.4 ± 112.4	154.3 ± 92.2	175.0 ± 119.5
Mushrooms	**0.048**	13.0 ± 12.5	12.8 ± 12.4	14.1 ± 12.2	15.1 ± 11.7
Coffee	0.472	256.9 ± 212.4	239.7 ± 150.7	246.9 ± 166.6	257.7 ± 212.6
Beef and pork	0.296	29.1 ± 19.2	28.9 ± 20.0	30.1 ± 20.9	33. ± 25.9
					Jonckheere-Terpstra test

## 4. Discussion

In this study, we investigated the interplay between sarcopenia, intake of the Japanese diet, and gut microbiota in a cohort of elderly individuals from the Kyotango region, recognised for their healthy longevity in Japan.^15^ To the best of our knowledge, this is the first report to clarify the association between gut microbiota and sarcopenia based on an analysis of Japanese dietary intake targeting a longevity region. The results of this study suggest that the abundance of bacteria associated with sarcopenia indicators is linked to Japanese dietary habits. In particular, several butyrate-producing genera belonging to the family *Lachnospiraceae* are key bacteria for sarcopenia prevention. Compared to prior studies, which mainly reported a reduced abundance of butyrate-producing bacteria in sarcopenic individuals, our study is unique in targeting a longevity region with distinct dietary traditions. By focusing on a region characterised by high consumption of traditional Japanese foods, our findings provide novel insights into host–microbiome interactions in healthy aging populations.

Specifically, our results showed that the abundance of an unclassified genus belonging to the family *Lachnospiraceae* was significantly lower and that of the genus *Megasphaera* was significantly higher in the sarcopenia group than in the non-sarcopenia group. The family *Lachnospiraceae* contains several butyrate-producing genera, including *Coprococcus* and *Lachnospira*. *Coprococcus* was positively correlated with gait speed, whereas *Lachnospira* had a weak positive correlation with grip strength in men. Although many of the correlations between gut microbiota and muscle-related indicators were relatively weak and lost statistical significance after FDR correction, a certain trends were observed.

The genus *Megasphaera*, the abundance of which increased in the sarcopenia group, is known to increase in athletes with low muscle strength and performance^30^ as well as in individuals with chronic inflammatory diseases, such as nonalcoholic hepatitis and genitourinary tract inflammation, and may be associated with the production of high levels of lipopolysaccharides.^31^^,^^32^

Regarding the relationship between dietary components and gut microbiota, our study established that consumption of the traditional Japanese diet in the healthy non-sarcopenia group resulted in an abundance of certain gut microbiota. Compared with the non-sarcopenia group, the sarcopenia group consumed the Japanese diet slightly less frequently; however, the difference in intake between the two groups was not significant. Hierarchical cluster analysis of Japanese food components and gut microbiota showed that the genera in the cluster with a high frequency of Japanese diet intake were primarily genera belonging to the family *Lachnospiraceae* and were consistent with the genera that showed a positive correlation with grip strength, gait speed, and SMI. These butyrate-producing bacteria were also positively correlated with protein, vitamin D, and dietary fibre intake in a hierarchical cluster analysis of nutritional components and gut microbiota.

Although the aetiology of sarcopenia remains unclear, a recent study has reported that dysbiosis allows inflammatory compounds of bacterial origin to enter the circulatory system and affect muscle metabolism.^33^ In animal studies, germ-free mice showed a decrease in skeletal muscle volume and muscle strength, while faecal microbial transplantation restored muscle mass in these mice.^34^ In addition, short-chain fatty acids, metabolites of the gut microbiota, have been reported to upregulate protein biosynthesis by activating the mTOR signalling pathway, thereby enhancing muscle volume and function,^35^ suggesting that several butyrate-producing bacteria are associated with sarcopenia and sarcopenia-related factors. We also found that the abundance of butyrate-producing bacteria in faeces was higher in those who consumed the Japanese diet more frequently. Recently,Han et al. revealed that butyrate concentration in faeces is significantly correlated with skeletal muscle volume in humans,supporting our results.^11^ Butyrate, a histone deacetylase inhibitor, reduces age-related muscle atrophy, oxidative stress, and intramuscular fat accumulation in mouse models.^36^ It also promotes regulatory T cell production, exhibits anti-inflammatory effects, and may help suppress hypercatabolism in sarcopenia.^37^ Additionally, exercise has been linked to increased levels of butyrate-producing bacteria, while a lack of exercise reduces these bacteria and restores the gut microbiota to its original state.^38^

The type of dietary intake that helps to prevent sarcopenia remains a key consideration. Adequate protein intake has been recognised to prevent sarcopenia in the elderly owing to their reduced protein sensitivity and synthesis capacity.^18^^,^^19^^,^^39^ However, we found no significant difference in protein intake between patients with and without sarcopenia. The gut microbiota-mediated effects of a high-protein diet are not always favourable for muscles.^40^^,^^41^ Mice fed a high-protein diet showed decreased Firmicutes/Bacteroides ratio and expense of gut microbiota that produce metabolic modulators such as short-chain fatty acids.^42^ Furthermore, it increases the abundance of pathogenic bacteria such as *Enterobacteriaceae,* leading to negative effects on muscle metabolism, such as the induction of inflammation and increased insulin resistance. Similar results have been obtained in human randomised controlled trials, where long-term administration of beef protein supplements to athletes reduced the abundance of beneficial faecal microbiota taxa, such as the genera *Bifidobacterium*, *Roseburia*, and *Blautia*.^43^ Notably, our study revealed that the abundance of the genus *Coprococcus,* which was positively correlated with physical function, was significantly correlated with the Japanese dietary components of seaweeds, beans, fish, green and yellow vegetables, and mushrooms. Fish and beans are rich in proteins and are known to increase butyrate-producing bacteria.^44^ Furthermore, taurine in fish and isoflavones in soybeans contribute to longevity.^45^ Our study revealed that healthy elderly Kyotango people tend to consume more fish and beans and limit their meat consumption. Therefore, appropriate intake of high-quality protein in the Japanese diet may prevent sarcopenia.

Despite these intriguing findings, our study had some limitations. First, because the Kyotango area is a region with a long life and good health, the number of people with sarcopenia was very low. Hence, owing to the small number of sarcopenia cases, the statistical power and generalisability were limited. Second, this was a cross-sectional study of older adults who underwent physical examinations at a single region in Japan. Due to the potential for selection bias, geographic considerations should be considered for generalisation. Third, the metabolites produced by intestinal bacteria, including short-chain fatty acids, were not measured, and it is unclear whether the levels of butyrate and other short-chain fatty acids truly increased. Despite these limitations, our findings are exploratory and provide preliminary evidence that certain bacteria, such as *Coprococcus* and *Lachnospiraceae*, might be associated with preserved muscle function in the elderly. Future longitudinal and interventional studies are needed to confirm these findings and explore the potential causal relationships between diet, gut microbiota, and risk of sarcopenia.

In conclusion, our findings suggest that consumption of the traditional Japanese diet may contribute to the prevention of sarcopenia by increasing the abundance of butyrate-producing bacteria, mainly genera belonging to the family *Lachnospiraceae.*

## Data Availability

The data generated in this study are deposited in the Sequence Read Archive (SRA) of the NCBI database with accession number PRJNA914797 and are available from February 28, 2023.

## References

[1] Tsugane S. Why has Japan become the world’s most long-lived country: insights from a food and nutrition perspective. Eur J Clin Nutr. 2021;75:921–928. doi: 10.1038/s41430-020-0677-5. 32661353 PMC8189904

[2] Cordes T, Bischoff LL, Schoene D, Schott N, Voelcker-Rehage C, Meixner C, Appelles L, Bebenek M, Berwinkel A, Hildebrand C, et al. A multicomponent exercise intervention to improve physical functioning, cognition, and psychosocial well-being in elderly nursing home residents: a study protocol of a randomized controlled trial in the PROCARE (prevention and occupational health in long-term care) project. BMC Geriatr. 2019;19:369. doi: 10.1186/s12877-019-1386-6. 31870314 PMC6929376

[3] Nishikawa H, Shiraki M, Hiramatsu A, Hara N, Moriya K, Hino K, Koike K. Reduced handgrip strength predicts poorer survival in chronic liver diseases: a large multicenter study in Japan. Hepatol Res. 2021;51:957–967. doi: 10.1111/hepr.13679. 34057800

[4] Tabata H, Otsuka H, Shi H, Sugimoto M, Kaga H, Someya Y, Naito H, Ito N, Abudurezake A, Umemura F, et al. Effects of exercise habits in adolescence and older age on sarcopenia risk in older adults: the bunkyo health study. J Cachexia Sarcopenia Muscle. 2023;14:1299–1311. doi: 10.1002/jcsm.13218. 37055913 PMC10235900

[5] Zhou HH, Liao Y, Peng Z, Liu F, Wang Q, Yang W. Association of muscle wasting with mortality risk among adults: a systematic review and meta-analysis of prospective studies. J Cachexia Sarcopenia Muscle. 2023;14:1596–1612. doi: 10.1002/jcsm.13263. 37209044 PMC10401550

[6] Chen LK, Woo J, Assantachai P, Auyeung T, Chou M, Iijima K, Jang HC, Kang L, Kim M, Kojima T, et al. Asian working group for Sarcopenia: 2019 consensus update on sarcopenia diagnosis and treatment. J Am Med Dir Assoc. 2020;21:300–307.e2. doi: 10.1016/j.jamda.2019.12.012. 32033882

[7] Kitamura A, Seino S, Abe T, Nofuji Y, Yokoyama Y, Amano H, Nishi M, Taniguchi Y, Narita M, Fujiwara Y, et al. Sarcopenia: prevalence, associated factors, and the risk of mortality and disability in Japanese older adults. J Cachexia Sarcopenia Muscle. 2021;12:30–38. doi: 10.1002/jcsm.12651. 33241660 PMC7890144

[8] Barry DJ, Wu SSX, Cooke MB. The relationship between gut microbiota, muscle mass and physical function in older individuals: a systematic review. Nutrients. 2024;17:81. doi: 10.3390/nu17010081. 39796514 PMC11722951

[9] Lee YA, Song SW, Jung SY, Bae J, Hwang N, Kim HN. Sarcopenia in community-dwelling older adults is associated with the diversity and composition of the gut microbiota. Exp Gerontol. 2022;167:111927. doi: 10.1016/j.exger.2022.111927. 35981616

[10] Liu C, Wong PY, Barua N, Li B, Zhang N, Chow SKH, Yu J, Ip M, Cheung WH, Duque G, et al. From clinical to benchside: Lacticaseibacillus and Faecalibacterium are positively associated with muscle health and alleviate age-related muscle disorder. Aging cell. 2025;19:e14485. doi: 10.1111/acel.14485. PMC12073917 39829204

[11] Han DS, Wu WK, Liu PY, Yang Y, Hsu H, Kuo C, Wang T. Differences in the gut microbiome and reduced fecal butyrate in elders with low skeletal muscle mass. Clin Nutr. 2022;41:1491–1500. doi: 10.1016/j.clnu.2022.05.008. 35667265

[12] Silva R, Pizato N, Mata F, Figueiredo A, Ito M, Pereira MG. Mediterranean diet and musculoskeletal-functional outcomes in community-dwelling older people: a systematic review and meta-analysis. J Nutr Health Aging. 2018;22:655–663. doi: 10.1007/s12603-017-0993-1. 29806854 PMC12876290

[13] Matsuyama S, Sawada N, Tomata Y, Zhang S, Goto A, Yamaji T, Iwasaki M, Inoue M, Tsuji I, Tsugane S. Association between adherence to the Japanese diet and all-cause and cause-specific mortality: the Japan public health center-based prospective study. Eur J Nutr. 2021;60:1327–1336. doi: 10.1007/s00394-020-02330-0. 32676701 PMC7987617

[14] Shimizu A, Okada K, Tomata Y, Uno C, Kawase F, Momosaki R. Association of Japanese and Mediterranean dietary patterns with muscle weakness in Japanese community-dwelling middle-aged and older adults: post hoc cross-sectional analysis. Int J Environ Res Public Health. 2022;19:12636. doi: 10.3390/ijerph191912636. 36231936 PMC9566278

[15] Naito Y, Takagi T, Inoue R, Kashiwagi S, Mizushima K, Tsuchiya S, Itoh Y, Okuda K, Tsujimoto Y, Adachi A, et al. Gut microbiota differences in elderly subjects between rural city Kyotango and urban city Kyoto: an age-gender-matched study. J Clin Biochem Nutr. 2019;65:125–131. doi: 10.3164/jcbn.19-26. 31592207 PMC6769410

[16] Nishikawa H, Shiraki M, Hiramatsu A, Moriya K, Hino K, Nishiguchi S. Japan society of hepatology guidelines for sarcopenia in liver disease (1st edition): recommendation from the working group for creation of sarcopenia assessment criteria. Hepatol Res. 2016;46:951–963. doi: 10.1111/hepr.12774. 27481650

[17] Miki T, Eguchi M, Akter S, Kochi T, Kuwahara K, Kashino I, Hu H, Kabe I, Kawakami N, Nanri A, et al. Longitudinal adherence to a dietary pattern and risk of depressive symptoms: the furukawa nutrition and health study. Nutrition. 2018;48:48–54. doi: 10.1016/j.nut.2017.10.023. 29469019

[18] Asaoka D, Toda K, Yoshimoto S, Katsumata N, Odamaki T, Iwabuchi N, Tanaka M, Xiao J, Nishikawa Y, Nomura O, et al. Sex-Specific associations of gut microbiota composition with sarcopenia defined by the Asian working group for Sarcopenia 2019 consensus in older outpatients: prospective cross-sectional study in Japan. Nutrients. 2025;17:1746. doi: 10.3390/nu17101746. 40431485 PMC12114429

[19] Kawahara T, Inazu T, Mizuno S, Tominaga N, Toda M, Toyama N, Suzuki G. DPVD research group. anti-sarcopenic effects of active vitamin D through modulation of anabolic and catabolic signaling pathways in human skeletal muscle: a randomized controlled trial. Metabolism. 2025;168:156240. doi: 10.1016/j.metabol.2025.156240. 40158795

[20] Kondo Y, Aoki H, Masuda M, Nishi H, Noda Y, Hakuno F, Takahashi S, Chiba T, Ishigami A. Moderate protein intake percentage in mice for maintaining metabolic health during approach to old age. GeroScience. 2023;45:2707–2726. doi: 10.1007/s11357-023-00797-3. 37118349 PMC10651611

[21] Das A, Jawla N, Meena V, Gopinath SD, Arimbasseri GA. Lack of vitamin D signalling shifts skeletal muscles towards oxidative metabolism. J Cachexia Sarcopenia Muscle. 2024;15:67–80. doi: 10.1002/jcsm.13378. 38041597 PMC10834326

[22] Okamura T, Hamaguchi M, Mori J, Yamaguchi M, Mizushima K, Abe A, Ozeki M, Sasano R, Naito Y, Fukui M. Partially hydrolyzed guar gum suppresses the development of sarcopenic obesity. Nutrients. 2022;14(6):1157. doi: 10.3390/nu14061157. 35334814 PMC8955723

[23] Saji N, Tsuduki T, Murotani K, Hisada T, Sugimoto T, Kimura A, Niida S, Toba K, Sakurai T. Relationship between the Japanese-style diet, gut microbiota, and dementia: a cross-sectional study. Nutrition. 2022;94:111524. doi: 10.1016/j.nut.2021.111524. 34952361

[24] Yasuda T, Takagi T, Asaeda K, Hashimoto H, Kajiwara M, Azuma Y, Kitae H, Hirai Y, Mizushima K, Doi T, et al. Urolithin A-mediated augmentation of intestinal barrier function through elevated secretory mucin synthesis. Sci Rep. 2024;14:15706. doi: 10.1038/s41598-024-65791-x. 38977770 PMC11231190

[25] Bolyen E, Rideout JR, Dillon MR, Bokulich NA, Abnet CC, Al-Ghalith GA, Alexander H, Alm EJ, Arumugam M, Asnicar F, et al. Reproducible, interactive, scalable and extensible microbiome data science using QIIME 2. Nat Biotechnol. 2019;37:852–857. doi: 10.1038/s41587-019-0209-9. 31341288 PMC7015180

[26] Callahan BJ, McMurdie PJ, Rosen MJ, Han AW, Johnson AJ, Holmes SP. DADA2: high-resolution sample inference from Illumina amplicon data. Nat Methods. 2016;13:581–583. doi: 10.1038/nmeth.3869. 27214047 PMC4927377

[27] The National Health and Nutrition Survey in Japan, 2019. Ministry of Health, Labour and Welfare. 2020. Accessed February 28, 2024. https://www.mhlw.go.jp/stf/seisakunitsuite/bunya/kenkou_iryou/kenkou/eiyou/r1-houkoku_00002.html.

[28] Food supply and demand chart, 2022. Ministry of Agriculture, Forestry and Fisheries. Assessed February 28, 2024. https://www.maff.go.jp/j/zyukyu/fbs/attach/pdf/index-20.pdf.

[29] Basic Survey on Coffee Demand Trends, 2020. National Coffee Roasters Association of Japan. Assessed February 28, 2024. https://coffee.ajca.or.jp/pdf/data-inyouchosa-202306.pdf.

[30] Liang R, Zhang S, Peng X, Yang W, Xu Y, Wu P, Chen J, Cai Y, Zhou J, Loor JJ. Characteristics of the gut microbiota in professional martial arts athletes: a comparison between different competition levels. PLoS One. 2019;14:e0226240. doi: 10.1371/journal.pone.0226240. 31881037 PMC6934331

[31] Lennard K, Dabee S, Barnabas SL, Havyarimana E, Blakney A, Jaumdally SZ, Botha G, Mkhize NN, Bekker L, Lewis DA, et al. Microbial composition predicts genital tract inflammation and persistent bacterial vaginosis in South African adolescent females. Infect Immun. 2017;86:e00410-17. doi: 10.1128/IAI.00410-17. 29038128 PMC5736802

[32] He X, Ding L, Su W, Ma H, Huang H, Wang Y, Ren H. Distribution of endotoxins in full scale pharmaceutical wastewater treatment plants and its relationship with microbial community structure. Water Sci Technol. 2018;77:2397–2406. doi: 10.2166/wst.2018.162. 29893728

[33] Liu C, Cheung WH, Li J, Chow SK, Yu J, Wong SH, Ip M, Sung JJY. Understanding the gut microbiota and sarcopenia: a systematic review. J Cachexia Sarcopenia Muscle. 2021;12:1393–1407. doi: 10.1002/jcsm.12784. 34523250 PMC8718038

[34] Lahiri S, Kim H, Garcia-Perez I, Reza MM, Martin KA, Kundu P, Cox LM, Selkrig J, Posma JM, Zhang H, et al. The gut microbiota influences skeletal muscle mass and function in mice. Sci Transl Med. 2019;11:eaan5662. doi: 10.1126/scitranslmed.aan5662. 31341063 PMC7501733

[35] Liu C, Wong PY, Wang Q, Huang T, Cui C, Zhang N, Cheung WH. Short-chain fatty acids enhance muscle mass and function through the activation of mTOR signalling pathways in sarcopenic mice. J Cachexia Sarcopenia Muscle. 2024;15:2387–2401. doi: 10.1002/jcsm.13573. 39482890 PMC11634463

[36] Walsh ME, Bhattacharya A, Sataranatarajan K, Qaisar R, Sloane L, Rahman MM, Kinter M, Van Remmen H. The histone deacetylase inhibitor butyrate improves metabolism and reduces muscle atrophy during aging. Aging cell. 2015;14:957–970. doi: 10.1111/acel.12387. 26290460 PMC4693467

[37] Atarashi K, Tanoue T, Oshima K, Suda W, Nagano Y, Nishikawa H, Fukuda S, Saito T, Narushima S, Hase K, et al. Treg induction by a rationally selected mixture of Clostridia strains from the human microbiota. Nature. 2013;500:232–236. doi: 10.1038/nature12331. 23842501

[38] Allen JM, Mailing LJ, Niemiro GM, Moore R, Cook MD, White BA, Holscher HD, Woods JA. Exercise alters gut microbiota composition and function in lean and obese humans. Med Sci Sports Exerc. 2018;50:747–757. doi: 10.1249/MSS.0000000000001495. 29166320

[39] Moore DR, Churchward-Venne TA, Witard O, Breen L, Burd NA, Tipton KD, Phillips SM. Protein ingestion to stimulate myofibrillar protein synthesis requires greater relative protein intakes in healthy older versus younger men. J Gerontol A Biol Sci Med Sci. 2015;70:57–62. doi: 10.1093/gerona/glu103. 25056502

[40] Mu C, Yang Y, Luo Z, Zhu W. Temporal microbiota changes of high-protein diet intake in a rat model. Anaerobe. 2017;47:218–225. doi: 10.1016/j.anaerobe.2017.06.003. 28629947

[41] Liu X, Blouin JM, Santacruz A, Lan A, Andriamihaja M, Wilkanowicz S, Benetti P, Tomé D, Sanz Y, Blachier F, et al. High-protein diet modifies colonic microbiota and luminal environment but not colonocyte metabolism in the rat model: the increased luminal bulk connection. Am J Physiol Gastrointest Liver Physiol. 2014;307:G459–G470. doi: 10.1152/ajpgi.00400.2013. 24970777

[42] Kiilerich P, Myrmel LS, Fjære E, Hao Q, Hugenholtz F, Sonne SB, Derrien M, Pedersen LM, Petersen RK, Mortensen A, et al. Effect of a long-term high-protein diet on survival, obesity development, and gut microbiota in mice. Am J Physiol Endocrinol Metab. 2016;310:E886–E899. doi: 10.1152/ajpendo.00363.2015. 27026084

[43] Moreno-Pérez D, Bressa C, Bailén M, Hamed-Bousdar S, Naclerio F, Carmona M, Pérez M, González-Soltero R, Montalvo-Lominchar M, Carabaña C, et al. Effect of a protein supplement on the gut microbiota of endurance athletes: a randomized, controlled, double-blind pilot study. Nutrients. 2018;10:337. doi: 10.3390/nu10030337. 29534465 PMC5872755

[44] Asnicar F, Berry SE, Valdes AM, Nguyen LH, Piccinno G, Drew DA, Leeming E, Gibson R, Le Roy C, Khatib HA, et al. Microbiome connections with host metabolism and habitual diet from 1,098 deeply phenotyped individuals. Nat Med. 2021;27:321–332. doi: 10.1038/s41591-020-01183-8. 33432175 PMC8353542

[45] Yamori Y, Sagara M, Arai Y, Kobayashi H, Kishimoto K, Matsuno I, Mori H, Shimosawa T. Soy and fish as features of the Japanese diet and cardiovascular disease risks. PLoS One. 2017;12:e0176039. doi: 10.1371/journal.pone.0176039. 28430815 PMC5400241

